# Iatrogenic stroke caused by cerebral air embolism and acute reperfusion therapy using hyperbaric oxygen

**DOI:** 10.1259/bjrcr.20210201

**Published:** 2022-02-04

**Authors:** Torbjørn Austveg Strømsnes, Ine Røed, Hanna Strøm, Rajiv Advani, Donata Biernat, Hege Ihle-Hansen

**Affiliations:** 1 Stroke unit, Department of Neurology, Oslo University Hospital, Oslo, Norway; 2 Unit for hyperbaric medicine, Department of Anaesthesiology, Oslo University Hospital, Oslo, Norway; 3 Department of Radiology, Elverum Hospital, Elverum, Norway; 4 Neuroscience Research Group, Stavanger University Hospital, Stavanger, Norway; 5 Department of Radiology, Oslo University Hospital, Oslo, Norway

## Abstract

**Objective:**

Air embolisms are mostly iatrogenic and a rare yet dreaded complication following percutaneous procedures. Intravascular entrapment of air can result in occlusion of end arteries and subsequent tissue ischemia and infarction. Cerebrovascular occlusions caused by air embolisms are time-sensitive and an uncommon cause of ischemic stroke, warranting an alternative acute management and reperfusion strategy.

**Methods:**

During a CT-guided lung biopsy, the patient developed left-sided paresis and sensory deficits prior to loss of consciousness. CT revealed air in the aorta, both ophthalmic arteries and vessels in the right parietal region. The patient was swiftly air-lifted to the nearest hyperbaric oxygen chamber for an alternate emergency reperfusion therapy. The following eight days the patient received hyperbaric oxygen therapy and gradually improved. Nine days after symptom onset he was discharged with a minor left facial palsy.

**Conclusions:**

Cerebrovascular occlusions are critical events regardless of etiology. Air embolism is rare but potentially catastrophic and can occur during both percutaneous procedures and surgeries. Vigilance and knowledge of this potential complication are needed to rapidly provide beneficial treatment. That is, high flow oxygen and correct positioning pending hyperbaric oxygen therapy.

## Background

Air embolisms are mostly iatrogenic. They are a rare yet dreaded complication following percutaneous procedures with potentially devastating effects. Intravascular entrapment of air can result in occlusion of end arteries and subsequent tissue ischemia and infarction.^1^ Cerebrovascular occlusions caused by air embolisms are time-sensitive and an uncommon cause of ischemic stroke, warranting an alternative acute management and reperfusion strategy.^2,3^

## Case presentation

A 70-year-old male underwent a computed tomography (CT)-guided lung biopsy of a potentially malignant right-sided lower lobe pulmonary lesion. The patient had a previous history of hypertension and type two diabetes mellitus and diabetic nephropathy.

**Figure 1.**
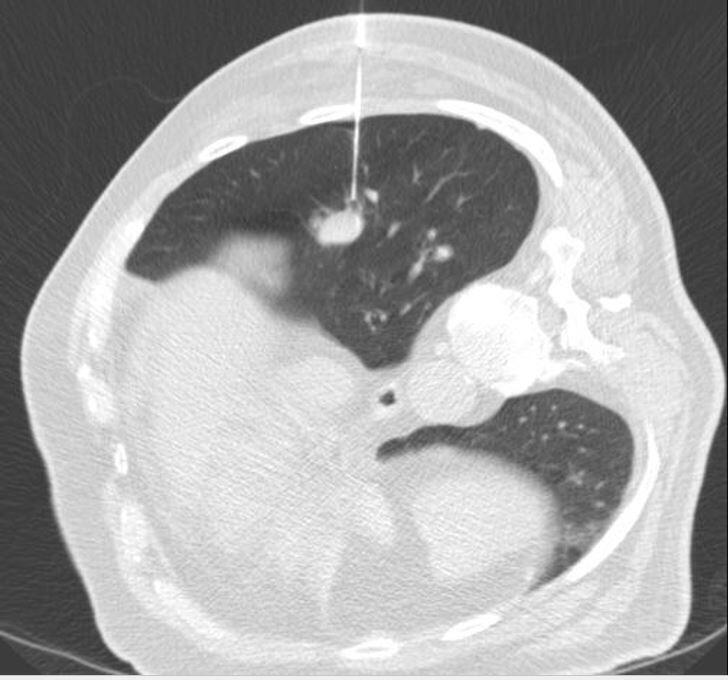
Chest CT during lung biopsy illustrating the biopsy needle and patient position during procedure.

Prior to the procedure, the patient was fasting and sedatives (5 mg Hydrocodone and 10 mg Oxazepam) were administered. The patient was encouraged to supress any coughing during the procedure to the best of his ability. The biopsy was performed in left lateral decubitus position using an 18G/22 mm aspiration needle (Figure 1).

During the initial biopsy, slight coughing and marginal haemoptysis was observed. This may transiently occur due to the introduction of blood into the bronchial tree. However, with the aspiration needle still in place, the coughing intensified. The aspiration needle was subsequently retracted, following which the patient developed left-sided sensorimotor symptoms followed by an abrupt loss of consciousness; Glasgow Coma Scale 3 (GCS 3).

**Figure 2.**
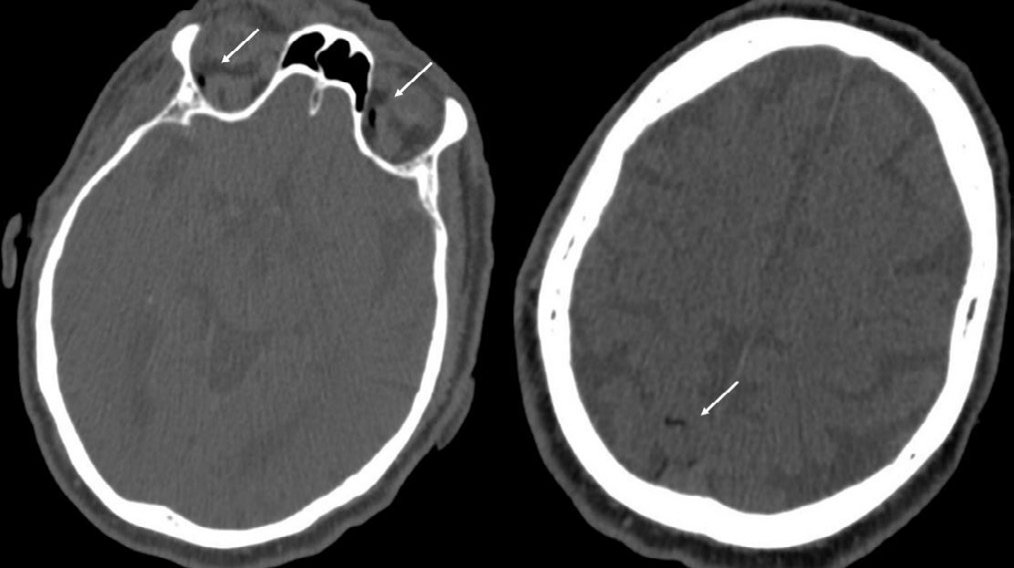
Acute head CT. Revealing air in the ophthalmic arteries on both sides and air in cortical vessels in the right parietal region. There are no signs of ischaemia at this time.

An acute chest CT was performed, showing no signs of pulmonary haemorrhage or pneumothorax. However, air bubbles were identified in the aorta. A subsequent head CT revealed air in both ophthalmic arteries, the right internal carotid artery, and cortical vessels in the right parietal region (Figure 2). These findings supported the suspicion of acute cerebrovascular symptoms due to arterial air emboli.
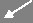


After the verification of air emboli, initial acute management was initiated. The patient was positioned in the right lateral decubitus position tilted with head down, aiming to contain air in the heart apex and avoid further air embolization, especially air reaching the coronary arteries, causing a subsequent heart attack. High flow oxygen was administered at 15 L/min. The patient was brought to the intensive care unit (ICU) for further monitoring and treatment. At the ICU, the patient regained consciousness and was observed to be disoriented; GCS 14.

After improvement on initial management, the patient was stable enough for transport and deemed eligible for hyperbaric oxygen therapy (HBO_2_T). Within one hour of symptom onset, the patient was air lifted to the nearest HBO_2_T treatment centre.

Upon arrival, the patient had an unchanged GCS of 14. Neurological examination revealed nystagmus, right sided facial palsy, drifting of left upper extremity and an inverted plantar reflex on the left side. Amounting to a National Institute of Health Stroke Scale (NIHSS) of 3. Vital parameters at arrival were pulse 100, oxygen saturation 100% (with 10 L/min on mask), blood pressure 167/75 mm Hg. The patient was brought without delay to the HBO_2_T chamber.

HBO_2_T was initiated according to a standardized algorithm (US Navy table 6) with initial recompression to 18 m sea water (MSW) (2,82 Atmospheres absolute (ATA)), then 9 MSW (1,92 ATA), for a total of 4 h and 50 min. The patient breathes 100% oxygen on mask at 20 min intervals, interrupted by 5 min of room air. After the initial oxygen period, the patient exhibited better orientation for time and place. After one hour of HBO_2_T, the patient presented fully reinstituted with no sensorimotor symptoms. After completion of the US Navy table 6 algorithm, the patients’ previous neurological symptoms were in complete remission and he had a GCS of 15.

**Figure 3.**
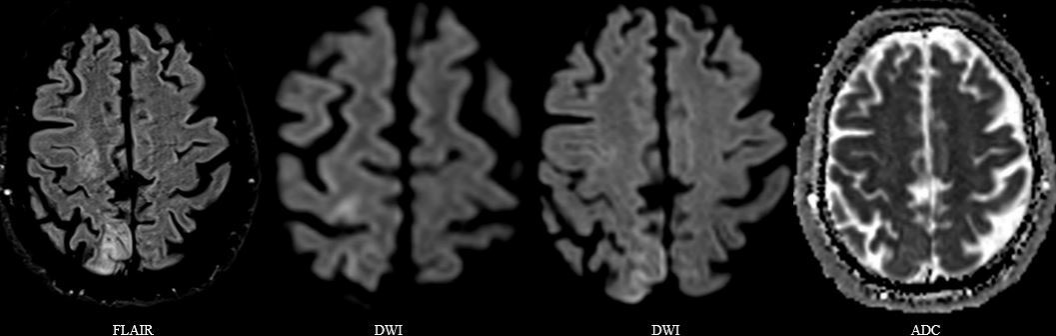
Cerebral MRI, with pathological T2-signals in the frontoparietal region and increased signal intensity on Diffusion-Weighted Images (DWI) and Apparent Diffusion Coefficient (ADC) representing vasogenic oedema. There is no visible infarcted tissue.

A few hours after completing the first session of HBO_2_T, the patient again experienced a recurrence of left-sided sensorimotor symptoms. One day after symptom onset, cerebral Magnetic Resonance Imaging (MRI) revealed vasogenic edema in the right periolandic^1^ and parietal region, without signs of tissue infarction (Figure 3). The decision to continue HBO_2_T was made, with shorter treatments as defined by US navy table 14/90, protocol defined as, 2 h recompression to 14 MSW (2,42 ATA), with three 30 min oxygen periods. This algorithm of HBO_2_T was repeated once daily for a total of eight days. During HBO_2_T period, there was minor fluctuations in symptoms, however an overall gradual reconstitution of neurological function. Nine days after symptom onset the patient was discharged from the hospital without any neurological, visual or other end organ sequalae apart from a marginal left sided facial palsy (NIHSS 1).

## Discussion

Symptomatic air emboli following percutaneous procedures is a rare, yet dreaded complication with high rates of severe morbidity and mortality.^4^ Air emboli can potentially get lodged in the heart and/or coronary arteries as well as smaller vessels both intra- and extra- cranially. Air emboli are mostly iatrogenic and occur due to the introduction of air into the pulmonary veins, arterial circulation, during cardiopulmonary bypass surgery, or due paradoxical embolization. Focal symptoms are caused by end artery occlusions.^1^ As illustrated here, the rapid recognition of stroke symptoms is crucial to facilitate timely treatment to ensure best clinical outcome. Recognition of stroke symptoms may potentially be delayed due to the use of periprocedural sedatives. The added benefit of a pulmonary CT angiography prior to a biopsy to minimize the risk of air emboli, should ideally be individually assessed. However, due to the rare nature of this complication and the numbers of biopsies performed each year, this is most likely not a cost-effective solution to implement for all patients.

The acute management aimed at preventing further dissemination of air emboli is crucial.^5^ When air is introduced in the pulmonary circulation, as in this case, positioning the patient on his right was a conscious choice. Had the air been introduced into the venous circulation (*e.g.,* via a central venous catheter), the correct position to minimize air dissemination would be on the patients left. These positioning maneuvres are meant to catch the circulating air prior to it leaving the heart. After the initial acute management, HBO_2_T to restore cerebral circulation to avoid permanent ischemic damage should be considered. Intravenous thrombolysis and/or endovascular thrombectomy are the gold standards of treatment for an acute ischemic stroke.^6,7^ These acute reperfusion therapies with thrombolytics or mechanical thrombectomy are not validated for the treatment of air emboli. However, we identified one case report successfully treating air embolus occlusion of the middle cerebral artery using endovascular aspiration.^8^ There are no data to our knowledge comparing endovascular versus hyperbaric treatment. In the present case, the emboli were multifocal and peripheral and thus deemed not suitable for an endovascular approach.

Hyperbaric Oxygen (HBO_2_T) immediately reduces the size of air emboli and creates a hyperoxic environment, which accelerates the egression of nitrogen from the bubbles. Furthermore, HBO_2_T increases oxygen delivery to the ischemic tissue. However, endothelial damage, inflammation, and cytotoxic oedema can cause clinical deterioration after initial improvement. HBO_2_T possesses antiinflammatory properties through inhibition of leukocyte adhesion, downregulation of proinflammatory cytokines thus leading to reduction of oedema.^9–11^ Sequential HBO_2_T is recommended until there is no further improvement.^12^

The literature on outcomes following arterial cerebral air emboli is sparse. The prognosis is assumed related to cause of emboli, the radiological extent of cerebral damage and time to HBO_2_T, where treatment initiated within eight hours is associated with a better prognosis.^2,13^ It is important to note that acute management including proper positioning and normobaric oxygen is crucial pending HBO_2_T. Clinicians performing percutaneous procedures and healthcare personnel monitoring patients after such a procedure should be vigilant for symptoms congruent with air embolization.

## Learning point

- All cerebrovascular occlusions are time-sensitive regardless of aetiology and warrant rapid diagnostics and knowledge of acute management and treatment options.
- In cases of cerebral air emboli HBO_2_T will replace our conventional acute reperfusion strategies.
- Correct patient positioning can minimise air dissemination.
- Clinicians performing percutaneous procedures and healthcare personnel monitoring patients after such a procedure should be vigilant for symptoms congruent with air embolization.

## Footnotes

* * periolandic region is the area of cerebral cortex in relation to the central sulcus and includes precentral gurus (primary motoric cortex) and the postcentral gurus (primary sensorimotoric cortex)

## References

[1] Muth CM, Shank ES. Gas embolism. N Engl J Med 2000; 342: 476–82. doi: 10.1056/NEJM200002173420706 10675429

[2] Tekle WG, Adkinson CD, Chaudhry SA, Jadhav V, Hassan AE, et al. Factors associated with favorable response to hyperbaric oxygen therapy among patients presenting with iatrogenic cerebral arterial gas embolism. Neurocrit Care 2013; 18: 228–33. doi: 10.1007/s12028-012-9683-3 22396189

[3] Judge C, Mello S, Bradley D, Harbison J. A systematic review of the causes and management of ischaemic stroke caused by nontissue emboli. Stroke Res Treat 2017; 2017: 7565702. doi: 10.1155/2017/7565702 29123937 PMC5662829

[4] Bessereau J, Genotelle N, Chabbaut C, Huon A, Tabah A, et al. Long-term outcome of iatrogenic gas embolism. Intensive Care Med 2010; 36: 1180–87. doi: 10.1007/s00134-010-1821-9 20221749

[5] Shaikh N, Ummunisa F. Acute management of vascular air embolism. J Emerg Trauma Shock 2009; 2: 180–85. doi: 10.4103/0974-2700.55330 20009308 PMC2776366

[6] Berge E, Whiteley W, Audebert H, De Marchis GM, Fonseca AC, et al. European stroke organisation (eso) guidelines on intravenous thrombolysis for acute ischaemic stroke. Eur Stroke J 2021; 6: I–LXII. doi: 10.1177/2396987321989865 PMC7995316 33817340

[7] Turc G, Bhogal P, Fischer U, Khatri P, Lobotesis K, et al. European stroke organisation (eso) - european society for minimally invasive neurological therapy (esmint) guidelines on mechanical thrombectomy in acute ischaemic strokeendorsed by stroke alliance for europe (safe). Eur Stroke J 2019; 4: 6–12. doi: 10.1177/2396987319832140 31165090 PMC6533858

[8] Belton PJ, Nanda A, Alqadri SL, Khakh GS, Chandrasekaran PN, et al. Paradoxical cerebral air embolism causing large vessel occlusion treated with endovascular aspiration. J Neurointerv Surg 2017; 9(4): e10. doi: 10.1136/neurintsurg-2016-012535.rep 27455873

[9] Buras JA, Reenstra WR. Endothelial-neutrophil interactions during ischemia and reperfusion injury: basic mechanisms of hyperbaric oxygen. Neurol Res 2007; 29: 127–31. doi: 10.1179/016164107X174147 17439696

[10] Thom SR. Hyperbaric oxygen: its mechanisms and efficacy. Plast Reconstr Surg 2011; 127 Suppl 1: 131S-141S. doi: 10.1097/PRS.0b013e3181fbe2bf 21200283 PMC3058327

[11] Camporesi EM, Bosco G. Mechanisms of action of hyperbaric oxygen therapy. Undersea Hyperb Med 2014; 41: 247–52. 24984320

[12] Antonelli C, Franchi F, Della Marta ME, Carinci A, Sbrana G, et al. Guiding principles in choosing a therapeutic table for dci hyperbaric therapy. Minerva Anestesiol 2009; 75: 151–61. 19221544

[13] Beevor H, Frawley G. Iatrogenic cerebral gas embolism: analysis of the presentation, management and outcomes of patients referred to the alfred hospital hyperbaric unit. Diving Hyperb Med 2016; 46: 15–21. 27044457

